# A Temperature-Based Model for Estimating Monthly Average Daily Global Solar Radiation in China

**DOI:** 10.1155/2014/128754

**Published:** 2014-01-29

**Authors:** Huashan Li, Fei Cao, Xianlong Wang, Weibin Ma

**Affiliations:** ^1^CAS Key Laboratory of Renewable Energy, Guangzhou Institute of Energy Conversion, Chinese Academy of Sciences, Guangzhou 510640, China; ^2^University of Chinese Academy of Sciences, Beijing 100049, China; ^3^College of Mechanical and Energy Engineering, Jimei University, Xiamen 361021, China; ^4^State Key Laboratory of Multiphase Flow in Power Engineering, Xi'an Jiaotong University, Xi'an 710049, China

## Abstract

Since air temperature records are readily available around the world, the models based on air temperature for estimating solar radiation have been widely accepted. In this paper, a new model based on Hargreaves and Samani (HS) method for estimating monthly average daily global solar radiation is proposed. With statistical error tests, the performance of the new model is validated by comparing with the HS model and its two modifications (Samani model and Chen model) against the measured data at 65 meteorological stations in China. Results show that the new model is more accurate and robust than the HS, Samani, and Chen models in all climatic regions, especially in the humid regions. Hence, the new model can be recommended for estimating solar radiation in areas where only air temperature data are available in China.

## 1. Introduction

Solar radiation data are essential for designing solar energy devices. However, the measurement of solar radiation is not easily available due to the cost and techniques involved [1]. The limited coverage of the measurement indicates that there is a need to establish theoretical methods for estimating solar radiation. Among the methods developed, those based on empirical correlations using commonly measured meteorological elements have attracted great attention owing to lower data requirement and computation cost [2].

The widely used correlations for estimating solar radiation are mainly based on sunshine duration and air temperature. In fact, the models estimating solar radiation from sunshine duration are generally more accurate than those involving other meteorological observations [3–6]. However, sunshine duration is not as readily available as air temperature data at standard meteorological stations [7, 8]. So, it is meaningful to elaborate models that estimate solar radiation based on air temperature as an alternative.

Two common approaches estimating solar radiation from air temperature use the methods of Hargreaves and Samani [9] (HS) and Bristow and Campbell [10] (BC). Since the establishment of the two models, many investigations concerning the HS and BC models have been carried out on the improvement in prediction accuracy and general validity, which were reviewed in detail by Liu et al. [2]. The HS model is primarily intended for application in monthly calculation [11]. Although the BC model is superior to the HS model on daily global solar radiation calculation in some studies [2, 3, 12], however, it is not as good as the HS model in estimation of monthly average solar radiation [13, 14]. The report from Bandyopadhyay et al. [13] that estimates solar radiation for 29 stations across India showed that the HS model and its modifications (Annandale et al. [15], Samani [16], and Allen [11, 17] models) outperform the BC model in monthly calculation. Similarly, Meza and Varas [14] demonstrated that the revised HS correlation, namely, Allen [11, 17] model, has a larger coefficient of determination than the BC model based on the monthly measured data from 21 stations in Chile. In addition, the HS model has been widely used because of its simplicity, and it is recommended in FAO-56 for solar radiation estimation [2].

However, the performance of the HS and its modifications varies significantly in different locations [2, 9]. This limits the application of these models in a large country like China with diversities in climate and geography. The present work aims to propose a new simple and practical method that gives good estimates of monthly average daily global solar radiation from air temperature for all climatic regions. The performance of the proposed model is validated by comparing with the original HS model and its two modifications against the measured data at 65 meteorological stations in China using statistical error tests.

## 2. Data and Methodology

### 2.1. Meteorological Data

China has extensive territory with complex topography, and hence many different climates with distinct features were found [18]. According to the scheme proposed by Zheng et al. [19], China can be classified into four types of climate zones based on moisture in terms of two indicators, namely, annual aridity index (AAI, ratio of annual average precipitation and potential evapotranspiration) and precipitation (*P*, mm). The four types are humid (AAI ≤ 1.00 and *P* > 800–900 mm (for Northeast China and mountain regions west to Sichuan *P* > 600–650 mm)), semihumid (1.00 < AAI ≤ 1.50 and 400–500 < *P* ≤ 800–900 mm (for Northeast China 400 < *P* ≤ 600 mm)), semiarid (1.50 < AAI < 4.00 (for Qinghai-Tibet Plateau 1.50 < AAI < 5.00) and 200–250 ≤ *P* ≤ 400–500 mm), and arid regions (AAI ≥ 4.00 (for Qinghai-Tibet Plateau AAI ≥ 5.00) and *P* < 200–250 mm) [19].

The measured data of monthly average daily global solar radiation (*H*, MJ/m^2^), monthly average maximum temperature (*T*_max⁡_, °C), and minimum temperature (*T*_min⁡_, °C) at 65 meteorological stations in China from 1971 to 2000 are used in the present paper. These stations cover the four climate zones and have a diverse range in latitude and altitude with the annual mean temperature difference between 6.20°C and 16.08°C. The information of these stations is given in Table 1. Note that the Δ*T* in the table is according to the definition in (1) as follows.

### 2.2. Models

The HS model [9] is the first procedure that calculates global solar radiation from *T*_max⁡_ and *T*_min⁡_ and defined as follows:

(1) $$\begin{array}{c} \frac{H}{H_{o}}=a_{1}\Delta T^{0.5}, \end{array}$$

where Δ*T* = *T*_max⁡_ − *T*_min⁡_, *H*_*o*_ is monthly average daily extraterrestrial radiation (MJ/m^2^), and *a*_1_ is empirical coefficient.

Following Hargreaves and Samani's pioneer work, Samani [16] and Chen et al. [5] suggested the modifications in the form of (2) and (3), respectively,

(2) $$\begin{array}{c} \frac{H}{H_{o}}=\left(a_{2}+b_{2}\Delta T+c_{2}\Delta T^{2}\right)\Delta T^{0.5}, \end{array}$$

where *a*_2_, *b*_2_, and *c*_2_ are empirical coefficients. Consider

(3) $$\begin{array}{c} \frac{H}{H_{o}}=a_{3}\Delta T^{0.5}+b_{3}, \end{array}$$

where *a*_3_ and *b*_3_ are empirical coefficients.

The characteristic underlying equations (1)–(3) is that they explicitly account for solar radiation and air temperature and implicitly include the influence of relative humidity by means of Δ*T*, which is linearly related to relative humidity [9]. While these models succeed in some areas, the assumption in the HS model as well as its modifications could lead to a reduction in estimation accuracy in some conditions [16]. The HS model assumes that Δ*T* is directly related to the fraction of *H*_*o*_ received at the ground level. However, in fact, many other factors besides solar radiation, such as latitude, altitude, cloudiness, humidity, wind speed, precipitable water, aerosol, and proximity to a large body of water, can influence Δ*T* in a given location [11, 16].

Among these factors, precipitable water has a considerable effect on solar radiation and then affects Δ*T*, especially in humid regions. The ways that precipitable water in the atmosphere affects solar radiation can be found in Garrison [20]. On the other hand, precipitable water is closely related to ambient temperature and relative humidity [21]. In view of this, to improve estimation in simplicity, air temperature is added together with the relative humidity implicitly presented in the HS model to exert precipitable water's effects on calculating solar radiation, and the HS model is revised as follows.

(4) $$\begin{array}{c} \frac{H}{H_{o}}=\left(a_{4}+b_{4}T_{a}\right)\Delta T^{0.5}+c_{4}, \end{array}$$

where *T*_*a*_  is monthly average air temperature (°C) and defined as *T*_*a*_ = (*T*_max⁡_ + *T*_min⁡_)/2 and *a*_4_, *b*_4_, and *c*_4_ are empirical coefficients.

### 2.3. Calibration and Comparison

A common method to calculate global solar radiation that is used by many models is to first determine *H*_*o*_. In this paper, *H*_*o*_ is calculated according to Duffie and Beckman [22]. The empirical coefficients of the four models (1)–(4) are, respectively, calibrated against the measured data of *H*, *T*_max⁡_, and *T*_min⁡_ (in terms of Δ*T* and *T*_*a*_) together with the calculated *H*_*o*_ using a solver that minimizes the square error of estimation with an iterative process.

The models' performance is assessed by four common statistical indicators, namely, mean percentage error (MPE, %), mean bias error (MBE, MJ/m^2^), root mean square error (RMSE, MJ/m^2^), and Nash-Sutcliffe equation (NSE), calculated from the estimated and measured values of *H*. These indicators are the ones that are applied most commonly in comparing the models of solar radiation estimation [23, 24] and can be calculated as follows:

(5) $$\begin{array}{c} \text{MPE}=\frac{\left\{\sum_{i=1}^{n}\left[100\times \left(H_{ci}-H_{mi}\right)/H_{mi}\right]\right\}}{n},\\ \text{MBE}=\frac{\left[\sum_{i=1}^{n}\left(H_{ci}-H_{mi}\right)\right]}{n},\\ \text{RMSE}=\sqrt{\frac{\left[\sum_{i=1}^{n}{\left(H_{ci}-H_{mi}\right)}^{2}\right]}{n}},\\ \text{NSE}=1-\frac{\sum_{i=1}^{n}{\left(H_{mi}-H_{ci}\right)}^{2}}{\sum_{i=1}^{n}{\left(H_{mi}-H_{ma}\right)}^{2}}, \end{array}$$

where *H*_*ci*_ and *H*_*mi*_ are, respectively, the *i*th calculated and measured values (MJ/m^2^), *H*_*ma*_ is the average of the measured values (MJ/m^2^), and *n* is the number of observations.

## 3. Results and Discussion

**Figure 1:**
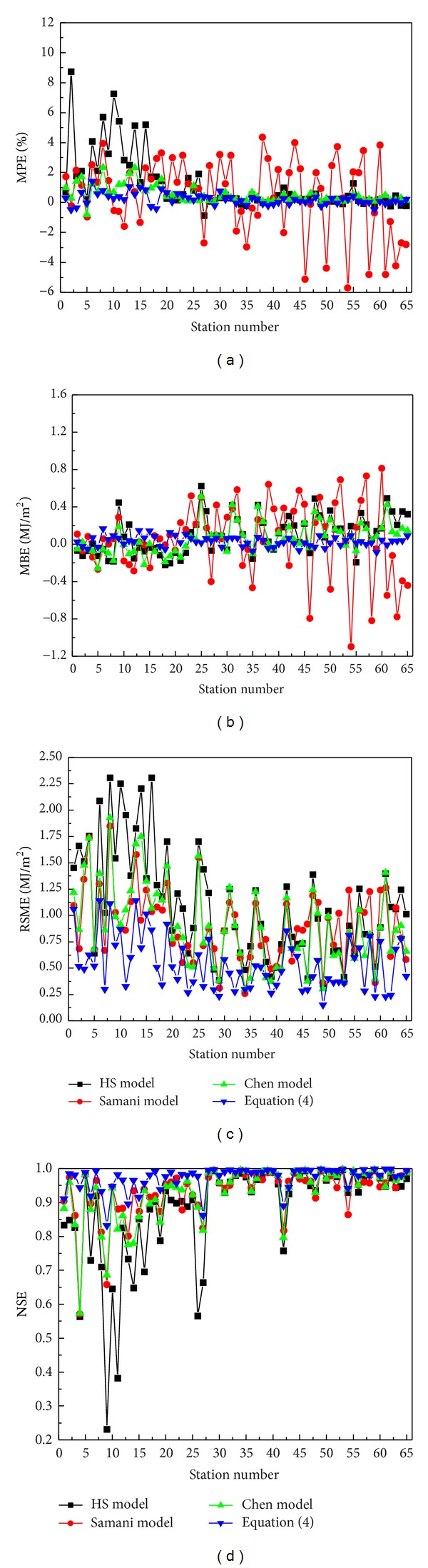
Statistical performance of the four models at the 65 stations (nos. 1–27 in the humid region, nos. 28–43 in the semihumid region, nos. 44–54 in the semiarid region, and nos. 55–65 in the aid region).

The empirical coefficients of the four models at each station are reported in Table 2. The table shows that the coefficients of the four models are site-dependent due to the use of local data bases. It should be mentioned that although a site-dependent model requires a data set with the measured *H* for determining the coefficients, this approach is frequently simpler to follow and may be more accurate than complicated mechanistic ones [25].  Figure 1 allows the values of MPE, MBE, RMSE, and NSE from the analysis of the measured and calculated *H* to be compared for the four models at the 65 stations, and the corresponding minimum, maximum, and average values of these statistical indicators are summarized in Table 3.

Figure 1 shows that the performance of these temperature-based models improves with Δ*T* in general, except for the Samani model in terms of MPE and MBE. Overall, the new model (4) produces more accurate estimates than the three existing models examined. This can be seen from the fact that (4) has smaller values of MPE, MBE, and RMSE and higher value of NSE compared with the others at all stations. The average values of MPE, MBE, RMSE, and NSE for (4) are 0.2199%, 0.0318 MJ/m^2^, 0.5408 MJ/m^2^, and 0.9724, respectively (in Table 3). Besides, the minimum value of NSE of (4) exceeds 0.80, which shows the superiority of the new model. Moreover, it is also found that compared with (4), the performance of the HS, Samani, and Chen models varies significantly in different climate regions.

**Figure 2:**
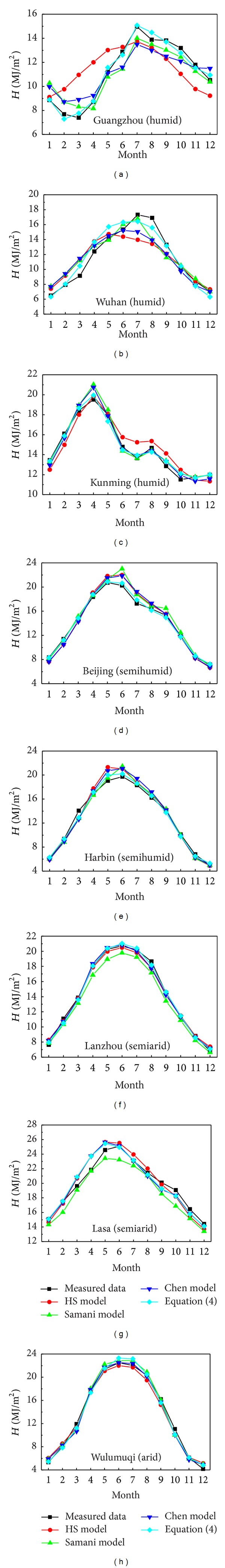
Comparison between the estimates of the four models and measured data at eight representative stations.

For clarity, the estimates of (4) and the three existing models are compared against the measured data at eight representative stations in Figure 2. These stations include Guangzhou (humid), Wuhan (humid), Kunming (humid), Beijing (semihumid), Harbin (semihumid), Lanzhou (semiarid), Lasa (semiarid), and Wulumuqi (arid) stations. As a rule of thumb, apart from the effects of topography, precipitable water in humid regions is generally larger than that in arid regions. Figure 2 shows that from the humid region to the arid region, the performance of the HS model and its modifications generally increases with Δ*T*. This fact also supports the Δ*T* sensitivity of the temperature-based models. The exception at Lasa station that deviates from the Δ*T* sensitivity results from the effects of altitude, which is in accordance with the results reported in the literature that the HS model and its modifications perform poorly at high-altitude sites [11, 13]. More importantly, it is interesting to find that the incorporation of *T*_*a*_ in the model can significantly relieve the sensitivity of Δ*T* and altitude associated with the temperature-based models. For example, the minimum NSE value of (4) at Kunming, Lanzhou, and Lasa stations with higher altitude is 0.9418 and at Guangzhou, Wuhan, and Kunming stations with lower Δ*T* is 0.9380. The two NSE values indicate that the new model can successfully account for the variation of *H* at sites having higher altitude or lower Δ*T*. Consequently, (4) is more robust than the three existing models examined here. Table 3 shows that the MPE, MBE, RMSE, and NSE of (4) range from −0.4718 to 1.4145%, from −0.0867 to 0.1681 MJ/m^2^, from 0.1524 to 1.1429 MJ/m^2^, and from 0.8324 to 0.9988, respectively. They are all the narrowest variation range for the statistical indicators among the four models.

Also, it can be found that with precipitable water increasing, namely, from the arid region to the humid region, the advantage of the new model over the HS, Samani, and Chen models becomes more prominent. In terms of NSE, (4) outperforms the HS, Samani, and Chen models approximately by 1.44%, 8.95% and 0.29% at Lasa station in the semiarid region whereas by 19.10%, 7.25%, and 11.40% at Wuhan station in the humid region. Note that the difference will be larger if Guangzhou station instead of Wuhan is used in the comparison, as evidently shown in Figure 2. Consequently, the modification of the HS model with the addition of *T*_*a*_ is reasonable.

## 4. Conclusions

This work stems from the fact that air temperature is commonly measured at many stations around the world, and the performance of the HS and its modifications varies significantly in different locations. To estimate monthly average daily global solar radiation from air temperature with better accuracy in all climatic regions, a new modification to the HS model is proposed. The new model is validated by comparing with the HS model and its two modifications against the measured data at 65 meteorological stations in China. The study demonstrates that the new model is more accurate and robust than the HS, Samani, and Chen models in all climatic regions, especially in the humid regions. Therefore, it can be recommended for estimating monthly average daily global solar radiation.

Admittedly, a limitation of this study is that the new model developed here is site-dependent, so when it is utilized in locations other than its based region, it is better to calibrate the empirical coefficients against the local data first. Future efforts should be directed to explore the correlation of the model's empirical coefficients with common factors and then develop a model for general application.

## Figures and Tables

**Table 1:** Information of the stations used in this study.

No.	Station	Lat. (°N)	Long. (°E)	Alt. (m)	Ave. Δ*T* (°C)	Climate [19]
1	Shanghai	31.40	121.48	6.0	6.20	Humid
2	Chongqing	29.58	106.47	259.1	6.41	Humid
3	Haikou	20.03	110.35	13.9	6.43	Humid
4	Shantou	23.40	116.68	2.9	6.53	Humid
5	Dalian	38.90	121.63	91.5	6.75	Humid
6	Nanchang	28.60	115.92	46.7	6.96	Humid
7	Chengdu	30.67	104.02	506.1	7.20	Humid
8	Changsha	28.22	112.92	68.0	7.22	Humid
9	Emeishan	29.52	103.33	3047.4	7.22	Humid
10	Guilin	25.32	110.30	164.4	7.29	Humid
11	Guangzhou	23.17	113.33	41.0	7.38	Humid
12	Guiyang	26.58	106.73	1223.8	7.45	Humid
13	Fuzhou	26.08	119.28	84.0	7.54	Humid
14	Nanning	22.63	108.22	121.6	7.55	Humid
15	Hangzhou	30.23	120.17	41.7	7.63	Humid
16	Ganzhou	25.85	114.95	123.8	7.72	Humid
17	Yichang	30.70	111.30	133.1	7.85	Humid
18	Mianyang	31.45	104.75	486.3	7.85	Humid
19	Wuhan	30.62	114.13	23.1	8.02	Humid
20	Hefei	31.87	117.23	27.9	8.08	Humid
21	Gushi	32.17	115.67	57.1	8.24	Humid
22	Nanjing	32.00	118.80	7.1	8.67	Humid
23	Mengzi	23.38	103.38	1300.7	9.98	Humid
24	Kunming	25.02	102.68	1892.4	10.51	Humid
25	Heihe	50.25	127.45	166.4	11.42	Humid
26	Lijiang	26.87	100.22	2392.4	11.56	Humid
27	Jinghong	22.00	100.78	582.0	11.57	Humid
28	Jinan	36.60	117.05	170.3	8.96	Semihumid
29	Tianjin	39.08	117.07	2.5	9.84	Semihumid
30	Xian	34.30	108.93	397.5	10.08	Semihumid
31	Changchun	43.90	125.22	236.8	10.47	Semihumid
32	Beijing	39.80	116.47	31.3	10.65	Semihumid
33	Shenyang	41.73	123.45	44.7	10.68	Semihumid
34	Zhengzhou	34.72	113.65	110.4	10.78	Semihumid
35	Juxian	35.58	118.83	107.4	11.06	Semihumid
36	Jiamusi	46.82	130.28	81.2	11.31	Semihumid
37	Harbin	45.75	126.77	142.3	11.49	Semihumid
38	Houma	35.65	111.37	433.8	12.58	Semihumid
39	Yanan	36.60	109.50	958.5	12.94	Semihumid
40	Chaoyang	41.55	120.45	169.9	13.33	Semihumid
41	Yushu	33.02	97.02	3681.2	14.85	Semihumid
42	Naqu	31.48	92.07	4507.0	14.93	Semihumid
43	Chengdu	31.15	97.17	3306.0	15.93	Semihumid
44	Aletai	47.73	88.08	735.3	12.04	Semiarid
45	Tongliao	43.60	122.27	178.5	12.13	Semiarid
46	Lanzhou	36.05	103.88	1517.2	12.19	Semiarid
47	Hailaer	49.22	119.75	610.2	12.22	Semiarid
48	Guyuan	36.00	106.27	1753.0	12.42	Semiarid
49	Taiyuan	37.78	112.55	778.3	13.10	Semiarid
50	Xilinhaote	43.95	116.07	989.5	13.24	Semiarid
51	Datong	40.10	113.33	1067.2	13.28	Semiarid
52	Xining	36.72	101.75	2295.2	13.39	Semiarid
53	Yining	43.95	81.33	662.5	13.83	Semiarid
54	Lasa	29.67	91.13	3648.7	14.32	Semiarid
55	Wulumiqi	43.78	87.65	935.0	10.30	Arid
56	Hetian	37.13	79.93	1374.5	12.30	Arid
57	Yinchuan	38.48	106.22	1111.4	12.74	Arid
58	Kashi	39.47	75.98	1288.7	12.80	Arid
59	Tulufan	42.93	89.20	34.5	13.23	Arid
60	Geermu	36.42	94.90	2807.6	13.90	Arid
61	Erlianhaote	43.65	111.97	964.7	14.23	Arid
62	Hami	42.82	93.52	737.2	14.92	Arid
63	Geer	32.50	80.08	4278.0	15.53	Arid
64	Ruoqiang	39.03	88.17	888.3	15.87	Arid
65	Dunhuang	40.15	94.68	1139.0	16.08	Arid

**Table 2:** Empirical coefficients of the four models at the 65 stations in China.

No.	Stations	HS	Samani			Chen		Equation (4)		
		*a*_1_	*a*_2_	*b*_2_	*c*_2_	*a*_3_	*b*_3_	*a*_4_	*b*_4_	*c*_4_
1	Shanghai	0.1610	1.0880	−0.2761	0.0203	−0.0320	0.4812	−0.0111	0.0010	0.3856
2	Chongqing	0.1028	0.0850	−0.0089	0.0016	0.2427	−0.3569	0.0734	0.0024	−0.0510
3	Haikou	0.1543	0.8163	−0.2148	0.0171	0.2027	−0.1231	−0.2070	0.0061	0.5269
4	Shantou	0.1588	0.3037	−0.0489	0.0040	0.2306	−0.1837	0.3433	0.0042	−0.7028
5	Dalian	0.1852	0.3425	−0.0434	0.0029	0.1448	0.1052	0.1552	0.0006	0.0597
6	Nanchang	0.1390	0.5998	−0.1712	0.0150	0.5675	−1.1313	0.4093	0.0013	−0.7748
7	Chengdu	0.1086	−0.2956	0.1030	−0.0064	0.2227	−0.3068	0.1165	0.0015	−0.0889
8	Changsha	0.1232	−0.5742	0.1820	−0.0117	0.4003	−0.7456	0.1615	0.0024	−0.2203
9	Emeishan	0.1503	0.0141	0.0254	−0.0009	0.3205	−0.4588	0.1563	−0.0029	0.0076
10	Guilin	0.1255	0.1556	−0.0366	0.0043	0.4751	−0.9459	0.4317	0.0005	−0.8567
11	Guangzhou	0.1278	−0.6600	0.1893	−0.0111	0.4454	−0.8640	0.4590	0.0022	−1.0344
12	Guiyang	0.1074	−0.6792	0.2013	−0.0128	0.2876	−0.4926	0.0677	0.0022	0.0096
13	Fuzhou	0.1321	1.0571	−0.2530	0.0172	0.3340	−0.5548	0.0209	0.0024	0.1684
14	Nanning	0.1351	−1.7651	0.4796	−0.0299	0.4274	−0.8042	0.3184	0.0032	−0.7019
15	Hangzhou	0.1296	−0.6555	0.2023	−0.0130	0.2336	−0.2872	0.0970	0.0011	0.0388
16	Ganzhou	0.1351	−0.1039	0.0302	0.0001	0.6017	−1.2976	0.4718	0.0012	−1.0032
17	Yichang	0.1197	−0.6137	0.1804	−0.0110	0.2701	−0.4218	−0.1108	0.0022	0.5341
18	Mianyang	0.1107	−0.1189	0.0525	−0.0029	0.1855	−0.2098	0.1030	0.0016	−0.0564
19	Wuhan	0.1285	2.1273	−0.4857	0.0294	−0.0494	0.5039	0.0520	0.0016	0.1414
20	Hefei	0.1312	0.7351	−0.1454	0.0087	0.0596	0.2036	0.0604	0.0007	0.1709
21	Gushi	0.1431	0.9199	−0.1792	0.0103	−0.0096	0.4388	0.0044	0.0010	0.3528
22	Nanjing	0.1332	1.1027	−0.2193	0.0123	0.0238	0.3224	0.0538	0.0006	0.2078
23	Mengzi	0.1465	−0.1047	0.0480	−0.0022	0.1948	−0.1528	0.1797	−0.0014	−0.0177
24	Kunming	0.1430	0.0348	0.0181	−0.0007	0.1983	−0.1807	0.1864	−0.0012	−0.0828
25	Heihe	0.1598	0.1533	0.0064	−0.0005	0.0418	0.3994	0.1448	−0.0011	0.0555
26	Lijiang	0.1589	0.0983	0.0040	0.0001	0.2920	−0.4541	0.2112	−0.0024	−0.0715
27	Jinghong	0.1351	0.1076	0.0072	−0.0004	0.0823	0.1816	0.1006	−0.0014	0.2315
28	Jinan	0.1493	0.2295	−0.0176	0.0010	0.1439	0.0163	0.1880	−0.0004	−0.0960
29	Tianjin	0.1543	−0.0542	0.0421	−0.0021	0.1631	−0.0275	0.1689	−0.0003	−0.0345
30	Xian	0.1225	0.0192	0.0208	−0.0010	0.1055	0.0539	0.0264	0.0007	0.2746
31	Changchun	0.1611	−0.5361	0.1377	−0.0067	0.1179	0.1401	0.1206	−0.0009	0.1492
32	Beijing	0.1560	−0.2924	0.0867	−0.0041	0.1284	0.0900	0.1472	−0.0008	0.0630
33	Shenyang	0.1472	−0.1274	0.0524	−0.0025	0.1635	−0.0533	0.1464	−0.0003	0.0100
34	Zhengzhou	0.1367	0.2959	−0.0268	0.0011	0.0701	0.2190	0.0701	0.0000	0.2193
35	Juxian	0.1413	0.3678	−0.0399	0.0017	0.0724	0.2297	0.0812	0.0003	0.1868
36	Jiamusi	0.1462	−0.0224	0.0331	−0.0016	0.0786	0.2274	0.0662	−0.0007	0.2783
37	Harbin	0.1492	−0.2103	0.0660	−0.0030	0.0921	0.1941	0.0808	−0.0004	0.2382
38	Houma	0.1296	0.5198	−0.0584	0.0022	0.0485	0.2877	0.0495	−0.0001	0.2883
39	Yanan	0.1276	0.4752	−0.0513	0.0019	0.0864	0.1486	0.0855	0.0002	0.1428
40	Chaoyang	0.1433	0.1449	−0.0001	0.0000	0.1400	0.0120	0.1085	−0.0003	0.1357
41	Yushu	0.1416	−0.0654	0.0246	−0.0007	0.2338	−0.3557	0.1959	−0.0003	−0.2057
42	Naqu	0.1426	0.1176	0.0014	0.0000	0.1981	−0.2147	−0.1381	−0.0031	1.0668
43	Chengdu	0.1360	0.4305	−0.0393	0.0013	0.2177	−0.3266	0.2212	0.0000	−0.3417
44	Aletai	0.1679	−0.5058	0.1160	−0.0049	0.1449	0.0800	0.2071	−0.0003	−0.1306
45	Tongliao	0.1479	−0.3715	0.0895	−0.0038	0.1124	0.1237	0.0921	−0.0005	0.2065
46	Lanzhou	0.1366	−0.5793	0.1132	−0.0045	0.2112	−0.2604	0.1530	0.0003	−0.0689
47	Hailaer	0.1642	0.2512	−0.0084	0.0001	0.0255	0.4852	0.1453	−0.0009	0.0662
48	Guyuan	0.1478	0.4469	−0.0526	0.0023	0.2455	−0.3442	0.1270	−0.0009	0.0951
49	Taiyuan	0.1361	0.0527	0.0144	−0.0006	0.1010	0.1274	0.0960	−0.0003	0.1554
50	Xilinhaote	0.1571	0.2633	−0.0112	0.0002	0.0295	0.4646	0.0966	−0.0006	0.2274
51	Datong	0.1474	−0.3479	0.0763	−0.0029	0.0975	0.1820	0.0963	−0.0003	0.1952
52	Xining	0.1469	0.1327	0.0001	0.0001	0.1986	−0.1893	0.0889	−0.0007	0.2290
53	Yining	0.1491	0.2021	−0.0066	0.0002	0.1175	0.1177	0.1081	0.0001	0.1501
54	Lasa	0.1696	0.5127	−0.0522	0.0019	0.2408	−0.2698	0.2120	−0.0003	−0.1509
55	Wulumuqi	0.1553	0.1945	−0.0121	0.0008	0.2261	−0.2280	0.0757	0.0010	0.2271
56	Hetian	0.1565	0.6063	−0.0725	0.0029	0.0564	0.3517	0.3131	−0.0017	−0.4670
57	Yinchuang	0.1613	0.4334	−0.0363	0.0012	−0.0032	0.5878	0.0095	−0.0005	0.5592
58	Kashi	0.1517	0.3119	−0.0261	0.0010	0.1455	0.0223	0.1281	0.0001	0.0789
59	Tulufan	0.1522	0.1287	0.0058	−0.0003	0.1190	0.1213	0.2036	−0.0006	−0.1536
60	Geermu	0.1770	−2.7052	0.4167	−0.0150	0.1885	−0.0427	0.0684	−0.0005	0.4149
61	Erlianhaote	0.1736	0.4895	−0.0400	0.0012	0.0461	0.4814	0.1089	−0.0009	0.2598
62	Hami	0.1640	0.2464	−0.0056	0.0000	0.0190	0.5611	0.1433	−0.0007	0.1118
63	Geer	0.1690	0.0684	0.0091	−0.0002	0.2882	−0.4701	0.2318	−0.0004	−0.2479
64	Ruoqiang	0.1489	0.4433	−0.0349	0.0010	0.0271	0.4865	0.1127	−0.0006	0.1754
65	Dunhuang	0.1550	0.5495	−0.0459	0.0013	0.0076	0.5923	0.0906	−0.0005	0.2816

**Table 3:** The minimum, maximum, and average values of the statistical indicators for the four models at the 65 stations in China.

Error	Model	Wet region			Semiwet region			Semi-arid region			Arid region			Overall		
		Min.	Max.	Ave.	Min.	Max.	Ave.	Min.	Max.	Ave.	Min.	Max.	Ave.	Min.	Max.	Ave.
MPE	HS	−0.8776	8.7307	2.5114	−0.2146	0.9753	0.2416	−0.1027	0.5700	0.2023	−0.2488	1.2711	0.1362	−0.8776	8.7307	1.1600
	Samani	−2.6927	3.9379	1.1221	−2.9563	4.3446	0.8382	−5.6928	4.0134	0.0326	−4.8029	3.8377	−0.9011	−5.6928	4.3446	0.5254
	Chen	−0.8200	2.3224	0.8990	0.0623	0.6648	0.2495	0.0623	0.5936	0.2474	0.0757	0.5148	0.2490	−0.8200	2.3224	0.5189
	Equation (4)	−0.4714	1.4145	0.4072	−0.1984	0.7587	0.1067	−0.2414	0.3613	0.1086	−0.4718	0.4250	0.0361	−0.4718	1.4145	0.2199

MBE	HS	−0.2237	0.6223	−0.0024	−0.1528	0.4181	0.1358	−0.0962	0.4891	0.1759	−0.1936	0.4919	0.2205	−0.2237	0.6223	0.0995
	Samani	−0.4043	0.5132	0.0285	−0.4681	0.6410	0.1842	−1.0949	0.6886	0.0646	−0.8158	0.8158	−0.0861	−1.0949	0.8158	0.0536
	Chen	−0.2592	0.5091	−0.0316	−0.1040	0.4187	0.1107	−0.0409	0.3427	0.1378	−0.0711	0.4193	0.1431	−0.2592	0.5091	0.0617
	Equation (4)	−0.0536	0.1681	0.0511	−0.0710	0.0747	0.0184	−0.0659	0.0934	0.0159	−0.0867	0.0919	0.0201	−0.0867	0.1681	0.0318

RMSE	HS	0.6398	2.3068	1.4972	0.3878	1.2721	0.7589	0.3489	1.3869	0.7508	0.5161	1.3956	0.9760	0.3489	2.3068	1.1009
	Samani	0.5503	1.8496	1.0371	0.2622	1.1235	0.7129	0.3624	1.2429	0.8792	0.3661	1.2611	0.9003	0.2622	1.8496	0.9074
	Chen	0.5085	1.9254	1.1351	0.2976	1.2655	0.6849	0.3005	1.2403	0.7090	0.3962	1.4056	0.8036	0.2976	1.9254	0.8961
	Equation (4)	0.2688	1.1429	0.6579	0.2356	0.8538	0.4382	0.1524	0.8140	0.4234	0.2301	0.8039	0.5204	0.1524	1.1429	0.5408

NSE	HS	0.2311	0.9837	0.7651	0.7575	0.9931	0.9540	0.9284	0.9960	0.9715	0.9297	0.9933	0.9656	0.2311	0.9960	0.8805
	Samani	0.5717	0.9818	0.8872	0.8169	0.9957	0.9616	0.8644	0.9969	0.9582	0.9424	0.9966	0.9690	0.5717	0.9969	0.9314
	Chen	0.5730	0.9817	0.8708	0.7962	0.9945	0.9616	0.9288	0.9968	0.9740	0.9470	0.9960	0.9761	0.5730	0.9968	0.9284
	Equation (4)	0.8324	0.9930	0.9534	0.8908	0.9975	0.9821	0.9418	0.9988	0.9889	0.9768	0.9987	0.9887	0.8324	0.9988	0.9724
